# Impact of a clinical pharmacist on medication safety in mental health Hospital-in-the-Home: a retrospective analysis

**DOI:** 10.1007/s11096-022-01409-4

**Published:** 2022-04-19

**Authors:** Mechaiel Farag, Kreshnik Hoti, Jeff Hughes, Leanne Chalmers

**Affiliations:** 1 Curtin Medical School, Faculty of Health Sciences, Curtin University, Kent Street, Bentley, WA 6102 Australia; 2 North Metropolitan Health Service—Mental Health Pharmacy, Brockway Road, Mount Claremont, WA 6010 Australia; 3 Division of Pharmacy, Faculty of Medicine, University of Prishtina, Prishtina, Kosovo

**Keywords:** Clinical pharmacist, Hospital-in-the-Home, Hospital-based home care, Medication safety, Medication reconciliation, Mental health

## Abstract

**Background:**

Integration of clinical pharmacists into multidisciplinary Mental Health Hospital-in-the-Home teams is increasing but little is known about the medication safety contribution these pharmacists make.

**Aim:**

To evaluate whether clinical pharmacist involvement in a Mental Health Hospital-in-the-Home service improved medication safety key performance indicators.

**Method:**

Medical records were retrospectively reviewed of all patients admitted to 2 Western Australian Mental Health Hospital-in-the-Home services from September to November 2015.

**Site 1:**

was a 16-bed service incorporating a clinical pharmacist as part of its multidisciplinary team.

**Site 2:**

was a similarly structured 18-bed service but without clinical pharmacist involvement. The primary outcome measure was completion of medication safety key performance indicators obtained from the Western Australian Government Pharmaceutical Review Policy and mental health-specific best practice guidelines.

**Results:**

Key performance indicators from *Site 1* (n = 75 records), which incorporated a clinical pharmacist, demonstrated significantly (*p* < 0.001) higher rates of completion of medication reconciliation [65 (87%) versus 17 (29%)], accurate adverse drug reaction list [73 (97%) versus 34 (58%)], accurate discharge medication list [51 (74%) versus 18 (45%)], accurate medication profile [74 (99%) versus 40 (68%)] and medication chart review [74 (99%) versus 0 (0%)] than *Site 2* (n = 59).

**Conclusion:**

Integrating a clinical pharmacist into a Mental Health Hospital-in-the-Home program significantly improved achievement of medication safety key performance indicators.

## Impact statements

- Integration of a clinical pharmacist into a Mental Health Hospital-in-the-Home service better facilitated the achievement of medication safety key performance indicators compared to a service without a pharmacist.
- Further research is required to optimise the clinical pharmacist’s role in patient counselling and prescribing support within a Mental Health Hospital-in-the-Home.
- With the rapid increase in implementation of Hospital-in-the-Home service models associated with the COVID-19 pandemic, these findings support integrating a clinical pharmacist in a Mental Health Hospital-in-the-Home program.

## Introduction

Hospital-in-the-Home (HiTH) is a care model where a patient is treated by hospital clinicians in the comfort of the patient’s home [1]. The HiTH model may also be known by other names, such as home (health)care teams [2] and home hospitalisation [3]. In this setting, as in all care settings, a range of medication-related problems (MRPs), errors and discrepancies can occur. In the specialised area of mental health (MH), MH-HiTH programs support the patient in all aspects of their treatment, including non-MH medical issues and psychosocial issues [4, 5]. Care is provided by a multidisciplinary team (MDT) consisting of a consultant psychiatrist, a psychiatric medical officer, a clinical nurse, a social worker, an occupational therapist and a clinical pharmacist (CP). An important part of the CP role is to optimise medication use and support patient adherence. Daily medication review, accurate adverse drug reaction (ADR) documentation, patient counselling and therapeutic drug monitoring (TDM) are some of the strategies employed by the CP in the HiTH setting to improve medication safety and contribute to better patient outcomes [6].

Another prominent CP task within the MH-HiTH setting is medication reconciliation on admission and discharge from the MH-HiTH program. HiTH is a critical point of transition of care [7], be it from hospital to home (via MH-HiTH), or from home to MH-HiTH, enabling the patient to be discharged earlier from a physical hospital bed or avoid physical hospitalisation, respectively. Unintentional changes to patients’ medication regimens often happen during such transitions of care [8]. Suboptimal communication between health professionals, and patients or care facilities can lead to medication errors and adverse drug events. Transition of care is the point where a large proportion of preventable medication-related adverse outcomes occur. Medication discrepancies, where there are differences between the medications the patient is prescribed and those they are actually taking, are especially common; these have been reported to affect 55.9% of patients [8]. A systematic review by El Morabet et al. found that between 5 and 87% of hospital readmission rates were caused by preventable MRPs [9], and that pharmacists were demonstrated to reduce this harm [10].

The benefits of clinical pharmacists in reducing medication-related harm have been clearly demonstrated across a range of healthcare settings [10–12], although most commonly in the hospital inpatient setting [13–15]. These benefits have included: reducing medication errors during transition of care; detecting and addressing MRPs; providing patients and their families/carers with verbal and written medication information to improve their engagement with, and outcomes of, their treatment; and optimisation of medication therapy in collaboration with the medication prescriber [16, 17], with recent evidence extending to psychiatric settings [18, 19]. As psychotropic medications are recognised as high risk medications [20], the Australian Commission on Safety and Quality in Health Care has recommended further adaptation of existing clinical pharmacy services to MH settings to improve medication safety in MH [21]. Although not explicitly defined in this context, medication safety could be defined as optimal use of medications, with appropriate TDM, so as to provide the best benefit, least side-effects and prevent medication-related harm [21].

With the integration of a CP, HiTH can provide important services across a variety of medical specialties, such as infectious diseases, cardiology and respiratory medicine [22–25]. MH-HiTH has evolved in recent times as a “non-traditional” practice setting for CPs. While there has been recent evidence to demonstrate the value of CP home visits on improving patient outcomes [26] and other robust evidence to demonstrate the utility of CP interventions in improving clinical outcomes in patients with severe and persistent mental illness [27], there is still a gap of published evidence relating specifically to the MH-HiTH setting [21], which involves elements of both home visits and other pharmacist interventions in people with mental illness. Hence this study was undertaken to compare achievement of medication safety key performance indicators (KPIs) between 2 similar MH-HiTH programs: 1 incorporating a CP and 1 without.

### Aim

This study aimed to evaluate whether CP integration within an MH-HiTH program improved measures of patient safety, by focussing on achievement of medication safety KPIs.

### Ethics approval

Ethics approval was obtained from the North Metropolitan Health Service—Mental Health, Public Health and Dental Services Human Research Ethics Committee (RGS0000000186) and Curtin University (HRE2017-0498).

## Method

### Study design

This retrospective cohort study involved review of patient case notes from comparable, government-subsidised MH-HiTH programs at 2 separate sites. *Site 1* was a 16-bed MH-HiTH program which had an integrated CP within its MDT and, for the purposes of this study, was considered the “intervention” arm. *Site 2*, the “control” arm, was an 18-bed MH-HiTH program that did not have any CP involvement. In each program, the patient was visited at least once daily by an MH-HiTH clinician, including a psychiatrist, psychiatric medical officer or nurse, to monitor their mental state and adjust medications where necessary. Intended length of stay (LoS) was 14 days, with some flexibility according to clinical response and patient choice. Any precipitating psychosocial factors were referred to appropriate services for longer-term follow-up. The 2 sites also had the same governance structure, policies and procedures, KPIs, as well as similar patient demographics, diagnoses and level of illness acuity, size and clinician-to-patient ratios. At both sites, all patients were between the ages of 18 and 65 years.

In *Site 1* only, the CP visited the patient once during their MH-HiTH admission to perform a medication review at the patient’s home. The CP had extensive inpatient MH clinical pharmacy experience and clinical pharmacy postgraduate qualifications. During the MH-HiTH admission, the CP also conducted medication reconciliation on admission and discharge, reviewed the medication chart daily, documented any ADRs and provided patient counselling and therapeutic drug monitoring (TDM), as well as prescribing support for doctors and medication information support for other clinicians. All patients had unlimited access to the CP via telephone during their admission.

Based on the assumption that medication reconciliation would be undertaken for 90% of patients receiving care at *Site 1* and 40% at *Site 2*, a sample size calculation estimated that 18 patients in each group would be statistically adequate for 95% power at the 95% confidence level [28].

### Data collection

Case notes of all patients admitted to each site from 1 September 2015 to 30 November 2015 (n = 120 at each site) were requested from the hospitals’ medical records departments. This 3-month period was chosen as it was soon after formation of each MH-HiTH program and alignment of their KPIs. The same 3-month period was chosen for both sites to minimise confounding.

Data were collected in August 2017 by tabulating the KPIs listed in Table 1 using Microsoft Excel^®^ 2013 (Microsoft Corporation, Redmond, Washington, USA). To minimise bias, 2 experienced MH CPs simultaneously collected the data, with 1 CP being independent of direct service provision. Patient characteristics, including gender, LoS and source of admission were recorded, and activities were recorded as having occurred (Yes) or not (No). Records were reviewed chronologically at each site. If the complete patient paper record (commonly known as patient case notes) was missing or unavailable at the time of data collection, it was excluded. If the patient paper record was present but incomplete, hospital electronic records were used to obtain relevant information wherever possible.

**Table 1:** Medication safety key performance indicators

KPI^a^ type	KPI name	Definition
Pharmaceutical Review Policy	1. Medication reconciliation on admission	Presence of documentation to demonstrate the performance of medication reconciliation on admission/transfer to the MH-HiTH^b^ program
	2. Medication reconciliation using more than 1 source	Presence of documentation demonstrating more than 1 source was consulted during medication reconciliation (e.g. GP^c^ list and community pharmacy history)
	3. Current medication profile documented	Presence of documentation demonstrating an accurate medication profile was kept current for the patient during their MH-HiTH admission
	4. Daily medication chart review	Presence of documentation demonstrating the medication profile was reviewed daily
	5. Provision of patient medication information	Presence of documentation demonstrating the patient was provided verbal and/or written medication information
	6. Presence of a medication list in the discharge summary	Presence of a medication list in the discharge summary filed in the notes
	7. The medication list in the discharge summary matches the discharge script	Absence of discrepancies between the discharge summary medication list and the prescription written on discharge from MH-HiTH
Mental Health KPIs	8. Discharged on multiple psychotropic medications	The discharge summary indicates the patient was prescribed more than 1 psychotropic medication from any particular class concurrently, e.g. more than 1 antipsychotic^d^
	9. Prescribed high dose psychotropic	The discharge summary indicates the patient was prescribed a psychotropic medication above its maximum licensed dose as listed in MIMS^e^
Patient Safety KPIs	10. Adverse drug reactions list documented	Presence of documentation of the patient’s adverse drug reaction list (or that there are no known drug allergies)
	11. Prescribed a medication listed on that patient’s adverse drug reaction list	Documentation indicating that during the MH-HiTH admission (or on the discharge prescription), the patient was prescribed a medication listed in their adverse drug reaction list

^a^KPI: key performance indicator

^b^MH-HiTH: mental health Hospital-in-the-Home

^c^GP: general practitioner

^d^As referred to in the Maudsley Prescribing Guidelines in Psychiatry [31]

^e^MIMS: an Australian official medication information database [32]

### Outcome measures

The primary outcome measure was achievement of medication safety KPIs, as detailed in the 2007 *WA Health* Pharmaceutical Review Policy [29], which was current at the time of admission of patients to this study. These KPIs remain relevant as, when the 2007 Policy was updated, the same KPIs were incorporated into its replacement, the *2019 WA Medication Review Policy* [16]. These KPIs were supported in the literature as surrogate outcomes for medication safety. For example, it is well-established in the literature that medication reconciliation reduces MRPs and, therefore, improves medication safety [30]. These KPIs are defined in Table 1. MH-specific KPIs were also evaluated; these included prevalence of psychotropic polypharmacy and high-dose psychotropic prescribing, as increasing prevalence of these is associated with increasing adverse effects, and clinically-appropriate minimisation is recognised as part of medication optimisation [31].

### Data analysis

Data were transferred into IBM SPSS^®^ Statistics Version 27 (IBM Corporation, Armonk, New York, USA) for analysis. Descriptive statistics were reported. Chi-square tests, with 1 degree of freedom, were used to assess the relationship between the integration of a CP in the MH-HiTH MDT and the medication safety KPIs. If chi-square test assumptions were not met, the Fisher’s Exact Test was used. A *p*-value of < 0.05 was considered statistically significant for all analyses.

## Results

During the study period, a total of 92 patients were admitted to *Site 1*, and 80 to *Site 2*. Due to missing or incomplete records, 17 records were excluded from *Site 1* and 21 from *Site 2*. This left 75 records eligible for analysis for *Site 1* and 59 for *Site 2*. The patients’ characteristics are summarised in Table 2. There were no statistically significant differences between the sites in terms of patient gender, LoS and admission source.

**Table 2:** Characteristics of the study patients

Characteristic	*Site 1* [n, (%)] (N = 75)	*Site 2* [n, (%)] (N = 59)	*p*-value
*Gender*			0.49
Male	25 (33%)	24 (41%)	
Female	50 (67%)	35 (59%)	
*Median LoS*^*a*^ *in days (IQR*^*b*^ *)*	14.0 (3)	14.0 (4)	0.146
*Admission source*			0.334
Community	25 (33%)	13 (22%)	
ED^c^	20 (27%)	20 (34%)	
Inpatient	30 (40%)	26 (44%)	

^a^LoS: length of stay

^b^IQR: interquartile range

^c^ED: emergency department

**Fig. 1:**
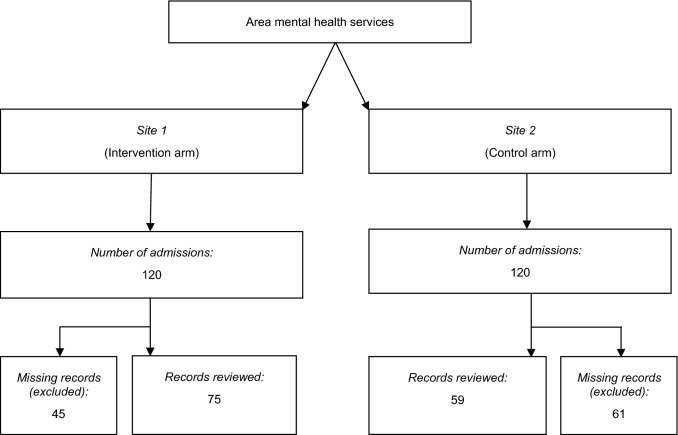
Flow chart of the data collection process

Overall, medication safety KPIs were achieved for a high proportion of patients in *Site 1*, though their completion was highly variable across the different activities for *Site 2*. There were statistically significant differences between Sites 1 and 2 in relation to documented medication reconciliation on admission (87% versus 29%), medication reconciliation using more than 1 source (83% versus 0%), complete medication profile (99% versus 68%), chart review (99% versus 0%), discharge medication list matching the script (74% versus 45%) and presence of an ADR list (97% versus 58%); all *p* < 0.001. Conversely, *Site 2* demonstrated a higher rate than *Site 1* in providing patient medication information (63% versus 21%, *p* < 0.001), and a lower rate of prescribing high dose psychotropics (7% versus 24%, *p* = 0.010). Figure 1 illustrates the data collection process, which is followed by the performance of each study site for each KPI as displayed in Table 3.

**Table 3:** Summary of the medication safety key performance indicators between the 2 sites

Medication Safety KPI^a^	*Site 1* n (%) (N^b^ = 75)	*Site 2* n (%) (N^b^ = 59)	*p*-value
1. Medication reconciliation on admission	65 (87)	17 (29)	< 0.001
2. Medication reconciliation using more than 1 source	62 (83)	0 (0)	< 0.001
3. Current medication profile documented	74 (99)	40 (68)	< 0.001
4. Daily medication chart review (*Site 2*: N = 58^c^)	74 (99)	0 (0)	< 0.001
5. Provision of patient medication information (*Site 1*: N = 73^c^)	15 (21)	37 (63)	< 0.001
6. Presence of a medication list in the discharge summary	74 (99)	58 (98)	1.000
7. The discharge summary’s medication list matches the discharge script (*Site 1*: N = 69; *Site 2*: N = 40)^d^	51 (74)	18 (45)	< 0.001
8. Discharged on multiple psychotropic medications (*Site 1*: N = 69; *Site 2*: N = 40)^e^	5 (7)	2 (3)	0.735
9. Prescribed high dose psychotropic (*Site 1*: N = 74; *Site 2*: N = 58)^e^	18 (24)	4 (7)	0.010
10. Adverse drug reactions list documented	73 (97)	34 (58)	< 0.001
11. Prescribed a medication listed on that patient’s adverse drug reaction list	5 (7)	0 (0)	0.067

^a^KPI: key performance indicator

^b^N: Total patient number for that site unless otherwise stated

^c^Patient(s) not taking any medication while admitted to HiTH so not required

^d^No script given to patient on discharge

^e^No medication-related information available in the case notes

## Discussion

### Statement of key findings

To our knowledge, this is the first study to evaluate the contribution of a CP to medication safety within an MH-HiTH program. While CP home visits improve patient outcomes [26] and CP interventions improve outcomes specifically in patients with severe and persistent mental illness [27], there is still no published evidence of the value of the CP in MH-HiTH setting, which involves various pharmacist interventions including home visits. This study found that the MH-HiTH program incorporating a CP had a higher rate of achievement of most medication safety KPIs evaluated than the MH-HiTH program without a CP. The higher rates of completion and documentation of medication safety KPIs in the MH-HiTH including the CP may be explained by the explicit focus of this role on medication management. While other clinicians can undertake medication safety activities, these are not their main clinical priority and they are not necessarily trained to undertake them in a comprehensive, systematic manner [11]. Therefore, inclusion of a CP in the MDT who was trained in the provision of CP services in a hospital setting (and could therefore translate them to this new clinical setting), who was familiar with the use of the medication management plan (MMP) [33], and whose role was solely focussed on medicines management, facilitated prioritisation of these activities.

### Strengths and weaknesses

A strength of this study was that simultaneous data collection by 2 experienced CPs reduced possible bias and ensured comprehensive review of patients’ medical records and KPIs. Additionally, the 2 services were very similar in their characteristics (as displayed in Table 2), apart from CP integration, imparting confidence that the differences observed were due to the CP’s involvement; however, unrecognised differences between the study cohorts cannot be completely discounted. While the study demonstrated strong associations between the presence of a CP on the MH-HiTH MDT and the achievement of medication safety KPIs, causality cannot be proven given the retrospective study design. A further weakness was reliance on documentation in patients’ medical records to collect the data, with the risk that certain tasks may have been performed but not documented. Another weakness is that medication safety was not directly assessed; instead, medication safety KPIs were used as surrogate measures for patient outcomes. While these data originated from 2015, they were collected during a unique time period, in which there was an opportunity to compare 2 similar MH-HiTH programs—one with a CP and the other without a CP. Shortly after the conclusion of this study, a CP was integrated within the *Site 2* MDT, where the CP role continues at that site. At the time of publication, both study sites remain largely unchanged in their model of service. Accordingly, this study’s data continue to offer important objective evidence of the benefit of a CP in the MDT, which has contributed to CP integration into all MH-HiTH services within the authors’ local health services.

### Interpretation

Importantly, this study highlighted the strong association between the presence of a CP and the achievement of medication safety KPIs at transitions of care, particularly medication reconciliation between the medication list in the discharge summary and the prescription on discharge. Tong et al. [34] showed in a randomised controlled trial (RCT) that integrating a CP within a general medical inpatient treating team, with the responsibility to complete a medication management plan within the discharge summary, reduced medication errors in those discharge summaries. During the study, *Site 1* utilised a similar procedure to that in Tong et al.’s study in that the MH-HiTH CP completed the medication sections of the discharge summary; this practice continues at the time of publication. While the level of agreement between the discharge medication list and discharge prescription at *Site 1* was 74%, and significantly higher than at *Site 2*, this was less than expected, potentially due to unplanned patient discharges on weekends, when there was no clinical pharmacy service available. This issue has subsequently been partially addressed by the MH-HiTH CP completing the discharge medication list 2 days before the planned discharge date then rechecking it on the day of discharge (if it is a weekday).

Despite evidence of significantly improved achievement of several medication safety KPIs at *Site 1*, there were some areas where CP integration did not appear to result in improved performance. For example, *Site 2* had a higher rate of provision of patient medication information. This may have reflected a difference in documentation, rather than completion of this activity, due to the presence of a checklist containing a check box for providing patient medication information in *Site 2* patient records. Furthermore, the CP’s competing work commitments precluded counselling of every patient during home visits, and provision of written patient medication information by other clinicians was inconsistently documented.

*Site 1* also had a significantly higher rate of high-dose psychotropic prescribing compared to *Site 2* (24% versus 7%, *p* = 0.010). This may be explained by the higher level of illness severity in *Site 1* or differences in the patients’ pre-admission medication history, although this was not evaluated in this study. It is recognised that severe, treatment-resistant mental illness may require the use of either high-dose or combination psychotropic therapy [31]. CPs have a potential role in supporting prescribers in the monitoring and potential rationalisation of high dose psychotropic therapy; this requires further investigation in the MH-HiTH setting.

On face value, the rate of prescribing of a medication listed on a patient’s ADR list at *Site 1* is a concerning finding. Upon further review, it was found that all 5 patients (7%) had no ill effects, as the ADR was of a mild nature, and the patient consented and was able to tolerate the rechallenge. For example, a patient whose ADR list stated “quetiapine causing sedation” agreed to retrial it at lower dose—the retrial was successful and the ADR documentation was revised to “quetiapine previously caused sedation on 25 mg nocte—tolerated rechallenge with 12.5 mg nocte”. This case highlights the importance of documenting the nature of the ADR, so if rechallenge is ever considered, it can be done judiciously. This further suggests that the CP actually improved prescribing by rationalising previously suboptimal ADR documentation.

### Further research

A recent systematic review by Abbott, et al. [26], reviewing RCTs from various settings but none relating to mental health, found no evidence that pharmacist home visits to patients at risk of medication-related problems improved hospital admission or mortality rates; Abbott, et al. remarked that *medication-related hospital admissions* would have been a more appropriate outcome measure. Yet, a more recent systematic review by Ng et al. [27] found that pharmacist-led interventions improve MH patient outcomes. Even though this systematic review searched for RCTs from all healthcare settings, none from MH-HiTH were presented in it. A future study could, therefore, investigate the effect of CP integration in a MH-HiTH program on patient outcomes, including medication-related hospital readmission rates and ED presentations, utilising the recently developed prescribing safety indicators specific to MH [35]. Werremeyer et al.’s recent review found that the most common factor associated with improving outcomes for patients with psychiatric and neurological conditions was incorporation of an MH CP into an MDT in predominantly inpatient, outpatient and clinic settings [36]. Future research could explore a novel approach to collect data on how a CP integrates into a MH-HiTH MDT to improve patient care by proactive discussion with MDT members, rather than making retrospective interventions. Given workload pressures, another future study could compare the efficiency of student pharmacists [37] and technicians [38] in conducting the medication reconciliation process.

## Conclusion

The MH-HiTH program incorporating a CP had statistically significant improvements in achievement of various medication safety KPIs compared to the program without a CP. Given the paucity of research in this area, this study provides an important contribution to understanding the role of a CP in the setting of MH-HiTH. With the current trend of increasing implementation of MH-HiTH programs, these findings support the value of CP integration as an important medication safety initiative. Future studies are needed to evaluate the impact of CP integration in this setting in improving patient outcomes, including reducing medication-related hospitalisation rates and ED presentations.
